# Adaptive Non-Singular Fast Terminal Sliding Mode Trajectory Tracking Control for Robotic Manipulator with Novel Configuration Based on TD3 Deep Reinforcement Learning and Nonlinear Disturbance Observer

**DOI:** 10.3390/s26010297

**Published:** 2026-01-02

**Authors:** Huaqiang You, Yanjun Liu, Zhenjie Shi, Zekai Wang, Lin Wang, Gang Xue

**Affiliations:** 1 School of Mechanical Engineering, State Key Laboratory of Advanced Equipment and Technology for Metal Forming, Key Laboratory of High-Efficiency and Clean Mechanical Manufacture of Ministry of Education, National Demonstration Center for Experimental Mechanical Engineering Education, Shandong University, Jinan 250061, China; 2 Institute of Marine Science and Technology, Shandong Key Laboratory of Intelligent Marine Engineering Geology, Environment and Equipment, Shandong University, Qingdao 266237, China

**Keywords:** robotic manipulator, Twin Delayed Deep Deterministic Policy Gradient algorithm, non-singular fast terminal sliding mode control, trajectory tracking control, nonlinear disturbance observer

## Abstract

This work proposes a non-singular fast terminal sliding mode control (NFTSMC) strategy based on the Twin Delayed Deep Deterministic Policy Gradient (TD3) algorithm and a nonlinear disturbance observer (NDO) to address the issues of modeling errors, motion disturbances, and transmission friction in robotic manipulators. Firstly, a novel modular serial 5-DOF robotic manipulator configuration is designed, and its kinematic and dynamic models are established. Secondly, a nonlinear disturbance observer is employed to estimate the total disturbance of the system and apply feedforward compensation. Based on boundary layer technology, an improved NFTSMC method is proposed to accelerate the convergence of tracking errors, reduce chattering, and avoid singularity issues inherent in traditional terminal sliding mode control. The stability of the designed control system is proved using Lyapunov stability theory. Subsequently, a deep reinforcement learning (DRL) agent based on the TD3 algorithm is trained to adaptively adjust the control gains of the non-singular fast terminal sliding mode controller. The dynamic information of the robotic manipulator is used as the input to the TD3 agent, which searches for optimal controller parameters within a continuous action space. A composite reward function is designed to ensure the stable and efficient learning of the TD3 agent. Finally, the motion characteristics of three joints for the designed 5-DOF robotic manipulator are analyzed. The results show that compared to the non-singular fast terminal sliding mode control algorithm based on a nonlinear disturbance observer (NDONFT), the non-singular fast terminal sliding mode control algorithm integrating a nonlinear disturbance observer and the Twin Delayed Deep Deterministic Policy Gradient algorithm (TD3NDONFT) reduces the mean absolute error of position tracking for the three joints by 7.14%, 19.94%, and 6.14%, respectively, and reduces the mean absolute error of velocity tracking by 1.78%, 9.10%, and 2.11%, respectively. These results verify the effectiveness of the proposed algorithm in enhancing the trajectory tracking accuracy of the robotic manipulator under unknown time-varying disturbances and demonstrate its strong robustness against sudden disturbances.

## 1. Introduction

As the core executive units of modern industrial automation systems, robotic manipulators have expanded their application scenarios from traditional structured environments such as welding processing [1] and material handling [2] to highly dynamic unstructured environments including surgical assistance [3], space exploration [4], and service robots [5]. During the execution of complex tasks, the end-effector of the robotic manipulator must accurately complete high-precision spatial movements along a predefined trajectory. However, robotic manipulators are highly complex multi-input multi-output systems characterized by strong nonlinearity, time-varying dynamics, and strong coupling [6]. Additionally, the structural parameters of the robotic manipulator are difficult to obtain accurately, and the dynamic model is partially unknown, resulting in significant uncertainties in the mathematical state-space model describing the motion of the robotic manipulators [7]. Functional configuration design and high-precision trajectory tracking control are effective approaches to address these challenges.

To better achieve trajectory tracking control of robotic manipulators, researchers have proposed various control methods, including fuzzy control [8,9], sliding mode control [10,11], neural network control [12,13], reinforcement learning control [14,15], and model predictive control [16,17].

Xian et al. [18] established a dynamic model for coordinated robotic manipulators using fuzzy set theory and developed an approximate constrained following servo control to ensure the consistent boundedness and consistent ultimate boundedness of the controlled system. They selected optimal parameters by solving fuzzy-based performance metrics. Zhang et al. [19] proposed a three-dimensional fuzzy active disturbance rejection controller for the mechanical arm of an iron roughneck by adding a three-dimensional fuzzy module to the classical ADRC. The controller output is adjusted based on tracking differential error, error rate of change, and error acceleration. Obadina et al. [20] developed a hybrid optimization algorithm for gray-box model identification of robotic manipulators and applied the model for real-time fuzzy trajectory tracking control. Jiang et al. [21] combined the Beetle Antenna Search (BAS) algorithm with Particle Swarm Optimization (PSO), introducing fractional calculus to dynamically adjust inertia weight and fractional order, achieving high-precision trajectory tracking control for robotic manipulators. However, fuzzy strategies overly rely on human experience, and parameter selection based on intelligent algorithms is prone to being trapped in local optima.

Sun et al. [22] employed radial basis function neural networks (RBFNN) to compensate online for uncertainty and unknown dynamics of system parameters. Chen et al. [23] optimized RBFNN parameters using an immune algorithm and designed adaptive rate compensation for unknown hysteresis errors similar to recoil and RBFNN approximation errors. However, the high computational complexity of neural network algorithms results in poor real-time tracking performance and presents physical implementation challenges. Carron et al. [24] combined inverse dynamics feedback linearization and data-driven error models with model predictive control. They processed offline data using Gaussian filtering and utilized extended Kalman filtering to estimate residual disturbances online, achieving bias-free tracking of the robotic manipulator. Kang [25] proposed an event-triggered model predictive control strategy for robotic manipulators with model uncertainty and input constraints. Trigger conditions were defined based on the weights of the predictive model and predicted tracking error. However, MPC is sensitive to system modeling accuracy, and its time-domain optimization mechanism only solves local optima within a finite time interval, making it difficult to ensure global asymptotic stability of the closed-loop system.

Sliding mode control has been widely applied in robotic manipulator control due to its simple physical implementation, strong robustness, and fast transient response [26]. Kali et al. [27] used a linear sliding mode surface for the trajectory tracking control of robotic manipulators, but this method only guarantees asymptotic convergence of the state [28]. Rapid convergence requires high gains, whereas terminal sliding mode control can ensure system convergence within finite time. Xu et al. [29] designed a discrete integral terminal sliding mode control law that incorporates delayed estimation of unknown disturbances and discretization errors in the robotic manipulator system and introduces an adaptive switching term to suppress sliding mode chattering effects. However, the chattering issue in sliding mode control can cause actuator damage or even system instability. To reduce or eliminate chattering, Makrini et al. [30] designed a variable boundary layer (BLT) sliding mode control method, which achieves joint safety and high-performance control by adjusting torque limit parameters and the expansion factor of the variable boundary layer.

Additionally, since certain system states are unmeasurable in practical applications, system uncertainty can affect transient performance and even lead to instability in robotic manipulator systems. To better achieve disturbance compensation, Fan et al. [31] proposed a fuzzy control strategy based on a spatial extended state observer to address the trajectory tracking control problem of robotic manipulator capturing a floating object in a microgravity environment. Yin et al. [32] proposed an adaptive non-singular terminal sliding mode control method based on NDO to observe the internal modeling errors of robotic manipulator and the external unknown time-varying disturbances acting on each joint for feedforward compensation. Zha et al. [33] estimated lumped uncertainties of the entire system using a hybrid observer composed of an adaptive time delay estimator based on gradient compensation and a second-order adaptive sliding mode observer. They then constructed an adaptive integral non-singular fast terminal sliding mode algorithm based on the backstepping method to stabilize the system and reduce chattering. Hu et al. [34] achieved real-time disturbance estimation and compensation by combining load torque estimation based on an improved harmonic drive flexibility model with friction compensation using a hybrid friction model, effectively improving trajectory tracking performance of the robotic manipulator under high-speed variable load conditions.

The aforementioned methods have achieved good results in robotic manipulators, but with the rapid development of artificial intelligence technology, data-driven machine learning approaches offer new insights for the intelligent tracking control of robotic manipulators. Reinforcement learning does not require the establishment of precise dynamic models, which makes it advantageous for solving sequential decision-making problems under highly nonlinear and uncertain conditions [35]. Viswanadhapalli et al. [36] utilized deep reinforcement learning control based on the Deep Deterministic Policy Gradient (DDPG) framework to achieve precise servo tracking of a flexible robotic manipulator, and evaluated the controller performance through hardware-in-the-loop (HIL) testing. Lu et al. [37] proposed an adaptive proportional-integral robust control method based on DDPG, which searches for the optimal controller parameters in a continuous action space using the dynamic information of the robotic manipulator. They also designed a reward function that combines a Gaussian function with Euclidean distance to ensure stable and efficient agent learning. Ren et al. [38] proposed an adaptive sliding mode control method based on DDPG reinforcement learning, leveraging RL autonomous learning capabilities to adaptively adjust the key parameters of the controller online. However, the DDPG strategy is susceptible to Q-value overestimation and insufficient exploration efficiency. The TD3 algorithm introduces a double Q-network, target policy noise, and delayed policy update techniques, achieving significant improvements in mitigating over-bias in action-value function estimation and enhancing policy stability. Zhu et al. [39] designed an adaptive sliding mode controller based on TD3 parameter optimization for variable-speed trajectory tracking of underactuated vessels in scenarios involving model uncertainty and external environmental disturbances. Fan et al. [40] addressed path tracking of unmanned underwater vehicles by integrating an improved experience replay strategy into TD3 while enhancing learning efficiency through refined regularization methods and dynamic reward functions, achieving faster convergence and superior tracking performance compared with mainstream classical DRL approaches.

Due to the highly nonlinear, multivariable strong coupling and the difficulty of accurately obtaining physical parameters, establishing an accurate dynamic model of robotic manipulators is extremely challenging. At the same time, the time-varying and uncertain nature of friction and external disturbances further complicates modeling. Although traditional control methods achieve satisfactory performance in known systems, their adaptability remains insufficient for unknown or uncertain systems. In order to address the trajectory tracking problem of robotic manipulator under unknown time-varying disturbances and modeling uncertainties, this paper proposes an improved NFTSMC method based on the TD3 algorithm and NDO. The main contributions are summarized as follows:

1. For tasks such as precision assembly and high-accuracy positioning that impose strict requirements on position and velocity control, a novel 5-DOF robotic manipulator configuration is designed, which reduces the end-effector load by positioning the joint motors at the front and simplifies dynamic model computation by introducing prismatic joint.
2. A nonlinear disturbance observer is employed to estimate internal modeling errors and external unknown time-varying disturbances of the robotic manipulator, followed by feedforward compensation. An improved nonsingular fast terminal sliding mode control law is designed based on boundary layer technique, and global system stability is analyzed using Lyapunov theory.
3. Adaptive control is achieved through DRL by proposing an adaptive NDONFT control method based on the TD3 algorithm, which employs a dual Q-network structure and selects the minimum Q-value to effectively mitigate value overestimation in DRL. The learning efficiency and stability of the agent are enhanced by modifying the reward function, thereby avoiding convergence to local optima.
4. Using the three joints of the designed 5-DOF robotic manipulator as examples, this paper verifies that the proposed method achieves excellent trajectory tracking performance and demonstrates stronger robustness against external unknown sudden disturbances.

The remainder of this paper is organized as follows: Section 2 presents the system principles and mathematical models of the robotic manipulator; Section 3 introduces the design process and stability analysis of the TD3NDONFT algorithm-based controller; Section 4 analyzes the simulation results; and Section 5 summarizes the main contributions of this paper.

## 2. System Principles and Mathematical Models

### 2.1. System Principles

**Figure 1:**
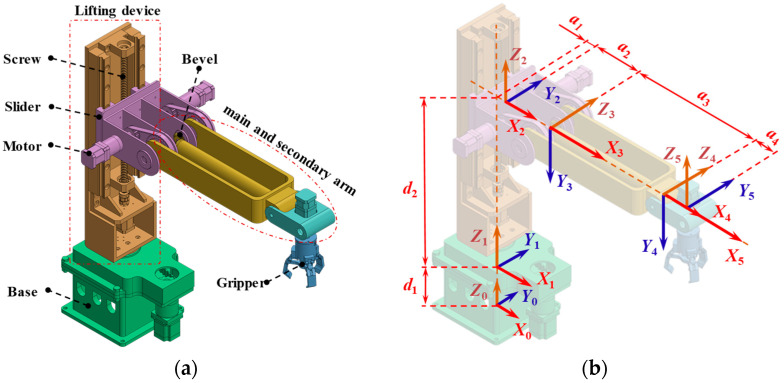
(**a**) Configuration of the robotic manipulator; (**b**) MDH coordinate system.

A 5-DOF robotic manipulator with a modular serial configuration is designed to meet the stringent position and velocity control requirements of precision assembly and high-accuracy positioning tasks, as shown in Figure 1a. The manipulator consists of a base, a lifting mechanism, a main arm, a secondary arm, and an end-effector gripper. The degrees of freedom of each joint are as follows: horizontal rotation of the base about a vertical axis (DOF1), linear translation of the lifting platform along a vertical rail (DOF2), pitch motion of the main arm about a horizontal axis (DOF3), independent pitch adjustment of the secondary arm about a parallel horizontal axis (DOF4), and rotation of the end-effector about a vertical axis (DOF5). DOF2 employs a screw-and-rail composite transmission mechanism, utilizing high-precision screw drives and low-friction rails to significantly enhance vertical positioning resolution and repeatability. The pitch joint of the secondary arm (DOF4) transmits power through two-stage bevel gear meshing, forming a front-mounted drive unit layout that effectively reduces end-effector inertia and enhances dynamic response capability. This arrangement accommodates high-speed, variable-acceleration trajectory tracking requirements. The overall symmetrical structure and compact transmission chain design reduce kinematic coupling effects and simplify the inverse kinematics model solution process. This configuration, through high-rigidity transmission, low-inertia drive, and decoupled motion chain design, lays the foundation for position closed-loop control in trajectory tracking.

In robotic manipulator modeling, the Denavit–Hartenberg (DH) method is a commonly used technique for describing the geometric structure and kinematic relationships of robotic manipulators. Compared to the standard DH method, the modified DH (MDH) method used in this study more accurately characterizes the geometric and kinematic features of the manipulator [41]. Table 1 lists the DH parameters of the manipulator based on the modified DH method. In this paper, the base coordinate system established using the MDH method is assumed to be the zero position, as shown in Figure 1b.

### 2.2. Dynamic Modeling

According to the Lagrange method, the dynamic model of an n-link robotic manipulator can be expressed as follows:

(1) $$M(q)\ddot{q}+C(q,\dot{q})\dot{q}+G(q)+F_{f}(\dot{q})=\tau +\tau_{d}$$

where $q,\dot{q},\ddot{q}\in R^{n}$ represent the joint position, velocity, and acceleration vectors of the robotic manipulator system, respectively. $M(q)\in R^{n\times n}$ is the symmetric positive definite inertia matrix of the system; $C(q,\dot{q})\in R^{n\times n}$ is the Coriolis and centrifugal force matrix; $G(q)\in R^{n}$ is the gravity vector; $F_{f}(\dot{q})\in R^{n}$ is the friction vector; $\tau_{d}\in R^{n}$ represents external time-varying disturbances. $\tau \in R^{n}$ is the torque input to the joints. Due to the complexity of the robotic manipulator structure, environmental variations, and measurement errors, it is generally difficult to obtain accurate values of $M(q),C(q,\dot{q}),G(q)$ in the dynamic equations. Therefore, $M(q),C(q,\dot{q}),G(q)$ is defined as:

(2) $$\left\{\begin{array}{l} M(q)=M_{0}(q)+\Delta M(q)\\ C(q,\dot{q})=C_{0}(q,\dot{q})+\Delta C(q,\dot{q})\\ G(q)=G_{0}(q)+\Delta G(q) \end{array}\right.$$

where $M_{0}(q),C_{0}(q,\dot{q}),G_{0}(q)$ represents the nominal value of the dynamic equation of the robotic manipulator, and $\Delta M(q),\Delta C(q,\dot{q}),\Delta G(q)$ represents the uncertainty term of the dynamic equation, i.e., the modeling error. Therefore, Equation (1) can be expressed as follows:

(3) $$M_{0}(q)\ddot{q}+C_{0}(q,\dot{q})\dot{q}+G_{0}(q)+\Delta E(q,\dot{q},\ddot{q})+F_{f}(\dot{q})=\tau +\tau_{d}$$

(4) $$\Delta E(q,\dot{q},\ddot{q})=\Delta M(q)\ddot{q}+\Delta C(q,\dot{q})\dot{q}+\Delta G(q)$$

where $\Delta E(q,\dot{q},\ddot{q})$ is the total modeling error of the robotic manipulator system. Setting $F(q,\dot{q},\ddot{q})=\tau_{d}-\Delta E(q,\dot{q},\ddot{q})-F_{f}(\dot{q})$ as the total disturbance of the system, including external disturbances, internal modeling errors, and friction. Equation (3) can be rewritten as:

(5) $$M_{0}(q)\ddot{q}+C_{0}(q,\dot{q})\dot{q}+G_{0}(q)=\tau +F(q,\dot{q},\ddot{q})$$

The purpose of this paper is to design a controller τ for the robotic manipulator system that enables the joint positions $q={\left(q_{1},q_{2},\cdots ,q_{n}\right)}^{T}$ and joint velocities $\dot{q}={\left({\dot{q}}_{1},{\dot{q}}_{2},\cdots ,{\dot{q}}_{n}\right)}^{T}$ of an n-DOF robotic manipulator to achieve high-precision tracking of the desired trajectory positions $q_{d}={\left(q_{d1},q_{d2},\cdots ,q_{dn}\right)}^{T}$ and velocities ${\dot{q}}_{d}={\left({\dot{q}}_{d1},{\dot{q}}_{d2},\cdots ,{\dot{q}}_{dn}\right)}^{T}$ under disturbances. To achieve this goal, the following assumptions [25,32] are made regarding system (1).

**Assumption 1.**  *For $\forall q\in R^{n}$, the symmetric positive definite inertia matrix M(q) is uniformly bounded, and there exists a normal constant $m_{1},m_{2}$ such that the following inequality holds:*

(6) $$m_{1}{\left\|x\right\|}^{2}\leq x^{T}M(q)x\leq m_{2}{\left\|x\right\|}^{2}$$

**Assumption 2.**  *For $\forall q\in R^{n}$, $\dot{M}(q)-2C(q,\dot{q})$ is a skew-symmetric matrix, it satisfies the following equation:*

(7) $$x^{T}\left[\dot{M}(q)-2C(q,\dot{q})\right]x=0,\forall x\in R^{n}$$

**Assumption 3.**  *In practice, for $\forall q\in R^{n}$, the gravity matrix satisfies, i.e., is always bounded.*

## 3. TD3NDONFT Controller Design

**Figure 2:**
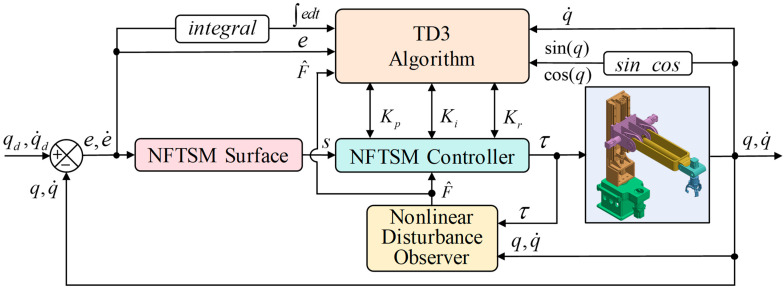
TD3NDONFT control algorithm principle diagram.

In order to achieve trajectory tracking control of robotic manipulators under complex environmental influences, this paper proposes a non-singular fast terminal sliding mode control method based on NDO and DRL, as shown in Figure 2. The NDO is used to estimate and compensate for the lumped disturbance, while the autonomous learning capability of DRL is employed to learn the controller parameters.

In the n-link robotic manipulator system, let the desired joint position, velocity, and acceleration be $q_{d},{\dot{q}}_{d},{\ddot{q}}_{d}$, respectively, and the actual joint position, velocity, and acceleration be $q,\dot{q},\ddot{q}$, respectively. Then, the tracking error and its derivative are defined as follows:

(8) $$\left\{\begin{array}{l} e=q_{d}-q\\ \dot{e}={\dot{q}}_{d}-\dot{q}\\ \ddot{e}={\ddot{q}}_{d}-\ddot{q} \end{array}\right.$$

The error state variables $\varepsilon_{1}$ and $\varepsilon_{2}$ are constructed based on the position error e, velocity error $\dot{e}$, and acceleration error $\ddot{e}$ of the robotic manipulator joints, that is $\varepsilon_{1}=e,\varepsilon_{2}={\dot{\varepsilon }}_{1}=\dot{e},{\dot{\varepsilon }}_{2}=\ddot{e}$. Then, the error dynamics equation of the manipulator can be written as:

(9) $$\left\{\begin{array}{l} {\dot{\varepsilon }}_{1}=\varepsilon_{2}\\ \begin{array}{c} {\dot{\varepsilon }}_{2}=-M_{0}^{-1}(q)\left[\tau -C_{0}(q,\dot{q})\dot{q}-G_{0}(q)+F(q,\dot{q},\ddot{q})\right]+{\ddot{q}}_{d} \end{array} \end{array}\right.$$

### 3.1. Design of Nonlinear Disturbance Observer

The disturbance is estimated by correcting the estimated value based on the difference between the estimated output and the actual output. Combining with Equation (5), the following nonlinear disturbance observer is designed:

(10) $$\begin{array}{ll} \dot{\hat{F}}(q,\dot{q},\ddot{q}) & =L(q,\dot{q})(F(q,\dot{q},\ddot{q})-\hat{F}(q,\dot{q},\ddot{q}))\\ & =-L(q,\dot{q})\hat{F}(q,\dot{q},\ddot{q})+L(q,\dot{q})(M_{0}(q)\ddot{q}+C_{0}(q,\dot{q})\dot{q}+G_{0}(q)-\tau ) \end{array}$$

where $L(q,\dot{q})$ is the gain matrix of the observer.

Since Equation (10) involves acceleration signals that cannot be accurately obtained by directly differentiating velocity signals in practical applications. This is because velocity signals inevitably contain sensor noise, quantization errors, and sampling jitter. Differentiation inherently amplifies high-frequency components, which significantly increases the influence of noise and results in severe distortion of the estimated acceleration. Furthermore, due to the high coupling of the manipulator system along with external disturbances, a nonlinear disturbance observer needs to be further designed to accurately estimate external disturbances in the manipulator system and feed them back to the controller for disturbance compensation.

Define auxiliary parameter variables as:

(11) $$z=\hat{F}(q,\dot{q},\ddot{q})-p(q,\dot{q})$$

where $p(q,\dot{q})$ is the designed function vector, according to [42], the gain matrix $L(q,\dot{q})$ and function vector $p(q,\dot{q})$ of the designed disturbance observer are given by:

(12) $$\left\{\begin{array}{l} L(q,\dot{q})=X^{-1}M_{0}^{-1}(q)\\ p(q,\dot{q})=X^{-1}\dot{q}\; \end{array}\right.$$

where X is an invertible matrix.

Substituting Equation (12) into Equation (11) and differentiating yields:

(13) $$\dot{z}=\dot{\hat{F}}(q,\dot{q},\ddot{q})-\dot{p}(q,\dot{q})=\dot{\hat{F}}(q,\dot{q},\ddot{q})-L(q,\dot{q})M_{0}(q)\ddot{q}$$

Substituting Equation (10) into Equation (13) yields:

(14) $$\begin{array}{cc} \dot{z} & =L(q,\dot{q})\left(M_{0}(q)\ddot{q}+C_{0}(q,\dot{q})\dot{q}+G_{0}(q)-\tau \right)-L(q,\dot{q})\hat{F}(q,\dot{q},\ddot{q})-L(q,\dot{q})M_{0}(q)\ddot{q}\\ & =L(q,\dot{q})\left(C_{0}(q,\dot{q})\dot{q}+G_{0}(q)-\tau \right)-L(q,\dot{q})\hat{F}(q,\dot{q},\ddot{q}) \end{array}$$

In summary, Equations (11)–(14) constitute the designed nonlinear disturbance observer.

In order to enable the nonlinear disturbance observer to accurately estimate disturbances, the linear matrix inequality (LMI) method is introduced to solve $L(q,\dot{q}),p(q,\dot{q})$. For convenience in derivation, the symbol is abbreviated as $L(q,\dot{q})=L,p(q,\dot{q})=p,$ $F(q,\dot{q},\ddot{q})=F$ in the following steps.

To prove that the observation error converges asymptotically, define the Lyapunov function as:

(15) $$V_{0}={\tilde{F}}^{T}X^{T}M_{0}(q)X\tilde{F}$$

where $\tilde{F}=F-\hat{F}$ is the observation error, $M_{0}(q)=M_{0}^{T}(q)>0$.

The derivative of the above Lyapunov function is:

(16) $${\dot{V}}_{0}={\dot{\tilde{F}}}^{T}X^{T}M_{0}(q)X\tilde{F}+{\tilde{F}}^{T}X^{T}{\dot{M}}_{0}(q)X\tilde{F}+{\tilde{F}}^{T}X^{T}M_{0}(q)X\dot{\tilde{F}}$$

According to Equations (11) and (14), the derivative of the observation error is:

(17) $$\begin{array}{ll} \dot{\tilde{F}} & =\dot{F}-\dot{\hat{F}}=\dot{F}-\dot{z}-\dot{p}\\ & =\dot{F}-L\left(C_{0}(q,\dot{q})\dot{q}+G_{0}(q)-\tau \right)+L\hat{F}-LM_{0}(q)\ddot{q}\\ & =\dot{F}+L\hat{F}-L\left(M_{0}(q)\ddot{q}+C_{0}(q,\dot{q})\dot{q}+G_{0}(q)-\tau \right)\\ & =\dot{F}+L\hat{F}-LF\\ & =\dot{F}-L\tilde{F} \end{array}$$

Generally, without prior knowledge of $\dot{F}$, it is assumed that the dynamic disturbance relative to the observer changes slowly [43], that is $\dot{F}=0$. Further, the observer error equation is obtained as:

(18) $$\left\{\begin{array}{l} \dot{\tilde{F}}=-L\tilde{F}=-X^{-1}M_{0}^{-1}(q)\tilde{F}\\ {\dot{\tilde{F}}}^{T}=-{\left(X^{-1}M_{0}^{-1}(q)\tilde{F}\right)}^{T}=-{\tilde{F}}^{T}M_{0}^{-T}(q)X^{-T} \end{array}\right.$$

Substituting Equation (18) into Equation (16) yields:

(19) $$\begin{array}{ll} {\dot{V}}_{0} & ={\dot{\tilde{F}}}^{T}X^{T}M_{0}(q)X\tilde{F}+{\tilde{F}}^{T}X^{T}{\dot{M}}_{0}(q)X\tilde{F}+{\tilde{F}}^{T}X^{T}M_{0}(q)X\dot{\tilde{F}}\\ & =-{\tilde{F}}^{T}M_{0}^{-T}(q)X^{-T}X^{T}M_{0}(q)X\tilde{F}+{\tilde{F}}^{T}X^{T}{\dot{M}}_{0}(q)X\tilde{F}-{\tilde{F}}^{T}X^{T}M_{0}(q)XX^{-1}M_{0}^{-1}(q)\tilde{F}\\ & =-{\tilde{F}}^{T}X\tilde{F}+{\tilde{F}}^{T}X^{T}{\dot{M}}_{0}(q)X\tilde{F}-{\tilde{F}}^{T}X^{T}\tilde{F}\\ & =-{\tilde{F}}^{T}\left(X-X^{T}{\dot{M}}_{0}(q)X+X^{T}\right)\tilde{F} \end{array}$$

Construct the following inequality:

(20) $$X+X^{T}-X^{T}{\dot{M}}_{0}(q)X\geq \gamma$$

where γ>0 is a symmetric positive definite matrix, there exists $\gamma^{\prime }>0$ such that the following equation holds:

(21) $${\dot{V}}_{0}\leq -{\tilde{F}}^{T}\gamma \tilde{F}=-\gamma^{\prime }V_{0}$$

It can be seen that the disturbance observer converges exponentially.

Since Equation (20) contains nonlinear terms, it must be converted to a linear matrix inequality to be solved. Define matrix $Y=X^{-1}$, then Equation (20) can be transformed into:

(22) $$Y^{T}+Y-{\dot{M}}_{0}(q)\geq Y^{T}\gamma Y$$

Since $\left\|{\dot{M}}_{0}(q)\right\|\leq \zeta$, then ${\dot{M}}_{0}(q)\leq \zeta I$, the sufficient condition for the above equation to hold is $Y^{T}+Y-\zeta I-Y^{T}\gamma Y\geq 0$, which is equivalent to:

(23) $$\left[\begin{array}{cc} Y^{T}+Y-\zeta I & Y^{T}\\ Y & \gamma^{-1} \end{array}\right]\geq 0$$

Based on the value of Y obtained from the solution, the observer gain matrix $L(q,\dot{q})$ and auxiliary variable $p(q,\dot{q})$ can be calculated.

### 3.2. NFTSM Controller Design

In traditional sliding mode control, the sliding surface is generally selected as a linear sliding surface. When the system state reaches the sliding surface, the linear sliding surface ensures that the error converges asymptotically to zero but does not guarantee finite-time convergence. In contrast, the terminal sliding surface guarantees that the error variable converges to zero within finite time.

Therefore, this paper adopts a non-singular fast terminal sliding mode surface [44]:

(24) $$r=\dot{e}+\alpha e+\beta {\left|e\right|}^{p/q}sign(e)$$

where α,β>0, p,q are all positive odd numbers, and p>q, sign(⋅) is the sign function. The derivative of Equation (24) with respect to time is as follows:

(25) $$\dot{r}=\ddot{e}+\alpha \dot{e}+\beta \frac{p}{q}{\left|e\right|}^{p/q-1}\dot{e}$$

Combining Equations (9) and (25) yields:

(26) $$\dot{r}=-M_{0}^{-1}(q)\left[\tau -C_{0}(q,\dot{q})\dot{q}-G_{0}(q)+F(q,\dot{q},\ddot{q})\right]+{\ddot{q}}_{d}+\alpha \dot{e}+\beta \frac{p}{q}{\left|e\right|}^{p/q-1}\dot{e}$$

Multiply both sides by the matrix $M_{0}(q)$ to obtain:

(27) $$M_{0}(q)\dot{r}=-\left[\tau -C_{0}(q,\dot{q})\dot{q}-G_{0}(q)+F(q,\dot{q},\ddot{q})\right]+M_{0}(q)\left[{\ddot{q}}_{d}+\alpha \dot{e}+\beta \frac{p}{q}{\left|e\right|}^{p/q-1}\dot{e}\right]$$

Because the acceleration vector $\ddot{q}$ is difficult to obtain in practice, and to avoid its appearance in the control law, define the variable:

(28) $$\left\{\begin{array}{l} {\dot{q}}_{r}=r+\dot{q}={\dot{q}}_{d}+\alpha e+\beta {\left|e\right|}^{p/q}sign(e)\\ {\ddot{q}}_{r}=\dot{r}+\ddot{q}={\ddot{q}}_{d}+\alpha \dot{e}+\beta \frac{p}{q}{\left|e\right|}^{p/q-1}\dot{e} \end{array}\right.$$

Substituting Equation (28) into Equation (27) yields:

(29) $$\begin{array}{ll} \tau & =M_{0}(q){\ddot{q}}_{r}+C_{0}(q,\dot{q})\dot{q}+G_{0}(q)-M_{0}(q)\dot{r}-F(q,\dot{q},\ddot{q})\\ & =M_{0}(q){\ddot{q}}_{r}+C_{0}(q,\dot{q}){\dot{q}}_{r}+G_{0}(q)-M_{0}(q)\dot{r}-C_{0}(q,\dot{q})r-F(q,\dot{q},\ddot{q}) \end{array}$$

According to the selected non-singular fast terminal sliding mode surface, the controller is formulated as follows:

(30) $$\left\{\begin{array}{l} \tau =\tau_{m}+\tau_{s}+\tau_{r}\\ \tau_{m}=M_{0}(q){\ddot{q}}_{r}+C_{0}(q,\dot{q}){\dot{q}}_{r}+G_{0}(q)\\ \tau_{s}=K_{p}r+K_{i}\int_{0}^{t}rdt\\ \tau_{r}=K_{r}sign(r) \end{array}\right.$$

$\tau_{m}$ is the equivalent controller obtained when the system reaches the sliding surface $r=\dot{r}=0$, and at this time the external disturbance is zero. $K_{p}$ and $K_{i}$ are the proportional gain and integral gain, respectively, with $K_{p}>0,K_{i}>0$. $K_{p}r$ is the sliding mode control term, which ensures that the system reaches stability. $K_{i}\int_{0}^{t}rdt$ is employed to further eliminate errors and external disturbances. $\tau_{r}$ is a robust controller primarily used to suppress external disturbances outside the nonlinear disturbance observer and internal model reconstruction errors, thereby enhancing disturbance rejection capability.

At the same time, by introducing a nonlinear disturbance observer to estimate the disturbances, the control law after adding its compensation is:

(31) $$\tau =\tau_{m}+\tau_{s}+\tau_{r}-\hat{F}=M_{0}(q){\ddot{q}}_{r}+C_{0}(q,\dot{q}){\dot{q}}_{r}+G_{0}(q)+K_{p}r+K_{i}\int_{0}^{t}rdt+K_{r}sign(r)-\hat{F}$$

From Equations (29) and (31), we can derive that:

(32) $$M_{0}(q)\dot{r}+C_{0}(q,\dot{q})r+K_{i}\int_{0}^{t}rdt=-K_{p}r-K_{r}sign(r)+\hat{F}-F$$

In order to limit the control signal to the feasible range of the actuator and avoid output overshoot, thereby preventing input saturation, a saturation function sat(r) is introduced to replace the sign function in the robust controller. The saturation function maintains linearity or smooth variation when the error is small, thereby reducing chattering, and gradually saturates when the error is large, thus preventing shocks caused by abrupt changes. The saturation function is defined as follows:

(33) $$sat(r)=\left\{\begin{array}{ll} sign(r) & \;\;\;\;if\frac{\left|r\right|}{h}\geq 1\\ \frac{\left|r\right|}{|r|+h}sign(r) & \;\;\;\;if\frac{\left|r\right|}{h}<1 \end{array}\right.$$

where h is a positive constant representing the thickness of the boundary layer.

### 3.3. Stability Analysis of the Control System and Proof of Finite-Time Convergence

In the stability analysis of control systems, we consider a Lyapunov function in integral form:

(34) $$V_{1}=\frac{1}{2}r^{T}M_{0}r+\frac{1}{2}{\left(\int_{0}^{t}rd\tau \right)}^{T}K_{i}\left(\int_{0}^{t}rd\tau \right)+{\tilde{F}}^{T}X^{T}M_{0}X\tilde{F}$$

Substituting Equation (15) into Equation (34), we obtain the derivative of $V_{1}$ with respect to time is:

(35) $${\dot{V}}_{1}=r^{T}\left[M_{0}(q)\dot{r}+\frac{1}{2}{\dot{M}}_{0}(q)r+K_{i}\left(\int_{0}^{t}rd\tau \right)\right]+{\dot{V}}_{0}$$

Considering the skew-symmetric property of the robotic manipulator dynamic equation, we have $r^{T}({\dot{M}}_{0}(q)-2C_{0}(q,\dot{q}))r=0$, and substituting Equation (32) into Equation (35), we derive:

(36) $$\begin{array}{cc} {\dot{V}}_{1} & =r^{T}[M_{0}(q)\dot{r}+C_{0}(q,\dot{q})r+K_{i}\int_{0}^{t}rd\tau ]+{\dot{V}}_{0}\\ & =r^{T}\left(-K_{p}r-K_{r}sign(r)+\hat{F}-F\right)+{\dot{V}}_{0}\\ & =r^{T}\left(-K_{p}r-K_{r}sign(r)-\tilde{F}\right)+{\dot{V}}_{0} \end{array}$$

Substituting Equation (21) into Equation (36) and simplifying yields ${\dot{V}}_{1}\leq -r^{T}K_{p}r-K_{r}\left\|r\right\|-\left\|r\right\|\left\|\tilde{F}\right\|-\gamma^{\prime }V_{0}\leq 0$. According to Lyapunov stability theory, the system state converges asymptotically to the sliding surface r=0. According to LaSalle’s invariance principle, when t→∞, r→0, that is, e→0, $\dot{e}\to 0$.

To prove that the system state converges to the sliding surface within a finite time, consider the Lyapunov function $V_{2}=\frac{1}{2}r^{T}M_{0}(q)r$, and differentiate it to obtain:

(37) $$\begin{array}{c} {\dot{V}}_{2}=r^{T}(-K_{p}r-K_{r}sign(r)-K_{i}\int_{0}^{t}rd\tau +\hat{F}-F)\\ \leq -r^{T}K_{p}r-r^{T}K_{r}sign(r)-r^{T}\tilde{F}\leq -K_{r}\left\|r\right\| \end{array}$$

Therefore ${\dot{V}}_{2}=\frac{dV_{2}}{dt}\leq -\sqrt{2}K_{r}{V_{2}}^{1/2}$, we obtain $dt\leq \frac{-dV_{2}}{\sqrt{2}K_{r}{V_{2}}^{1/2}}$, let $t_{r}$ denote the system convergence time, and $V_{2}(0)$ represent the initial state, at this time $V_{2}\left(t_{r}\right)=0$. Integrating the above yields:

(38) $$\int_{0}^{t_{r}}dt\leq \int_{V_{2}(0)}^{V_{2}(t_{r})}\frac{-dV_{2}}{\sqrt{2}K_{r}{V_{2}}^{1/2}}={\left.\sqrt{2}K_{r}{V_{2}}^{1/2}\right|}_{V_{2}(0)}^{V_{2}(t_{r})}$$

The convergence time is calculated as $t_{r}\leq \sqrt{2}\frac{{V_{2}}^{1/2}(0)}{K_{r}}$, The trajectory tracking error of the robotic manipulator system tends to zero within finite time.

When |r|≥h, the saturation function behaves as the sign function sign(r), which does not affect the convergence of the control law. When |r|<h, the convergence speed decreases to some extent, but r has been controlled within a certain range. The robotic manipulator system achieves the desired convergence by adjusting h, so the saturation function does not affect the final convergence properties of the system [34].

### 3.4. Deep Reinforcement Learning TD3 Adaptive Control

Reinforcement learning is a key branch of machine learning that obtains control strategies through interaction with the environment. RL problems are usually formulated as a Markovian decision process (MDP), which mainly includes the environment, agent, reward, state, and action [40]. Specifically, at each time step t, the robotic manipulator agent selects an action $a_{t}$ based on the current state $s_{t}$, and the environment provides a reward $r_{t}$ quantifying the performance of the robotic manipulator. The ultimate goal of RL is to find an effective control strategy π(s) that maximizes the cumulative long-term return $R_{t_{k}}=\sum_{t=t_{k}}^{\infty }\gamma^{t-t_{k}}r_{t}$, where $\gamma \in \left(0,1\right]$ is the discount factor indicating the importance of future rewards, and $t_{k}$ represents the initial time step.

TD3 is an advanced DRL method designed to address control problems with continuous action spaces. It employs an actor-critic architecture, where the critic evaluates the value function $Q_{\pi }(s_{t},a_{t})$ of actions and states. Specifically, at time step t, $Q_{\pi }(s_{t},a_{t})$ represents the long-term reward obtained by taking action $a_{t}$ under policy π(s). Under this formulation, the Q function satisfies:

(39) $$Q_{\pi }(s_{t},a_{t})=r_{t}+\gamma Q_{\pi }(s_{t+1},\pi (s_{t+1}))$$

The ultimate goal of DRL is to learn an optimal control strategy $\pi^{*}(s)$ through Equation (40) so that the value function is maximized.

(40) $$\pi^{*}(s)=\arg\underset{\pi }{\max}Q_{\pi }(s_{t},a_{t})$$

**Figure 3:**
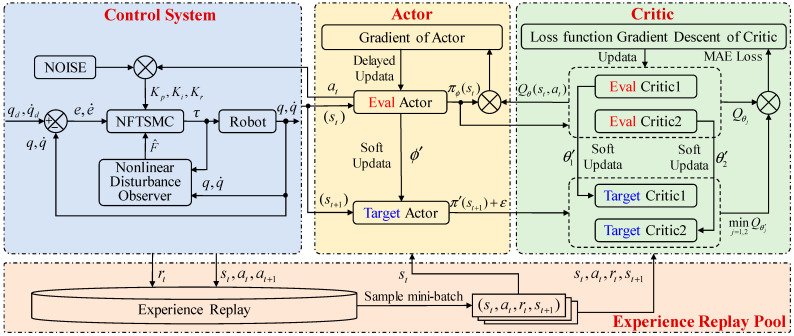
Trajectory tracking control framework for NFTSMC of robotic manipulators based on NDO and TD3.

Generally, neural networks (NNs) are employed to solve Equations (39) and (40) within the actor-critic architecture, as shown in Figure 3. TD3 mainly consists of six NNs. Specifically, two evaluate critic networks are used to estimate the Q-functions, $Q_{\theta_{1}}$ with parameter $\theta_{1}$ and $Q_{\theta_{2}}$ with parameter $\theta_{2}$. Additionally, an evaluate actor $Q_{\pi }$ with parameters ϕ is used for policy updates. Each actor and critic network are paired with a target network to ensure training stability, denoted by $Q_{{\theta^{\prime }}_{1}}$, $Q_{{\theta^{\prime }}_{2}}$ and $Q_{\pi^{\prime }}$. The update of the evaluate critics is expressed by the following loss function:

(41) $$L(\theta_{j})=\frac{1}{N}\sum_{i=1}^{n}\omega_{i}\delta_{i}^{2}$$

(42) $$\delta_{i}=r_{i}+\gamma \underset{j=1,2}{\min}Q_{{\theta^{\prime }}_{j}}(s_{i+1},\pi_{\phi^{\prime }}(s_{i+1})+\varepsilon )-Q_{\theta_{j}}(s_{i},\pi_{\phi^{\prime }}(s_{i}))$$

where N is the batch size selected transitions at each step, $\delta_{i}$ is known as the time differencing error (TD error), and $\omega_{i}$ is the importance sampling weight (ISW) of the *i*-th sample. The target value estimate is calculated by selecting the minimum estimated Q value to reduce the error overestimation problem in traditional Q learning. Additionally, to ensure smoother updates of the critics and prevent overfitting, a noise signal is added as a regularization term to the target actor, $\varepsilon ∼clip(N(0,\sigma ),-\bar{c},\bar{c})$, where σ is the noise standard deviation, N denotes a standard normal distribution, and $\bar{c}$ represents the noise clipping limit.

The evaluate actor updates by maximizing the learned Q-function and adopts the deterministic policy gradient for policy updates.

(43) $$\nabla_{\phi }J(\phi )=\frac{1}{N}\sum_{i=1}^{N}\nabla_{a}Q_{\theta }{\left(s_{i},a_{i}\right)}_{a=\pi_{\phi }(s_{i})}\cdot \nabla_{\phi }\pi_{\phi }(s_{i})$$

where $J(\phi )=(1/N)\sum_{i=1}^{N}Q_{\theta }(s_{i},a_{i})a=\pi_{\phi }(s_{i})$ is the expected return to be maximized, $\nabla_{a}Q_{\theta }(s_{i},a_{i})a=\pi_{\phi }(s_{i})$ is the gradient of Q-value with respect to action, and $\nabla_{\phi }\pi_{\phi }(s_{i})$ is the gradient with respect to the evaluate actor parameter ϕ. Additionally, to ensure a stable training process, the target network parameters are updated by soft updating to track the evaluated network.

(44) $$\begin{array}{l} \theta^{\prime }\leftarrow (1-\tau )\theta^{\prime }+\bar{\tau }\theta \\ \phi^{\prime }\leftarrow (1-\tau )\phi^{\prime }+\bar{\tau }\phi \end{array}$$

where $\bar{\tau }\in \left(0,1\right]$ is the soft factor that determines the update rate of the target networks. It is worth noting that the update frequencies of the actor and critic networks in TD3 are inconsistent. Generally, the critic network updates more frequently than the actor network to minimize the Q-value error before introducing policy updates.

#### 3.4.1. State Space Design

The state $s_{t}$ represents the environmental information perceived by the robotic manipulator agent. The objective is to design a controller that integrates sliding mode control based on disturbance observation compensation with TD3 to reduce the errors between the actual and target positions, as well as between the actual and desired velocities of the robotic manipulator. Based on the design of the NDONFT controller and considering the structural characteristics of the designed 5-DOF robotic manipulator, the first three joints are sufficient to cover most of the target workspace, while the two joints at the end are mainly used to adjust the posture of the actuator. Therefore, simulation is conducted on the three joints of the designed robotic manipulator, and the current state is defined as follows:

(45) $$\begin{array}{l} S_{1}=\{\sin q_{1},\cos q_{1},\sin d_{2},\cos d_{2},\sin q_{3},\cos q_{3},{\dot{q}}_{1},{\dot{d}}_{2},{\dot{q}}_{3}\}\\ S_{2}=\{e_{1},e_{2},e_{3},\int e_{1}dt,\int e_{2}dt,\int e_{3}dt\}\\ S_{3}=\{\sum a_{t-1},{\hat{F}}_{1},{\hat{F}}_{2},{\hat{F}}_{3}\} \end{array}$$

For $S_{1}$, the observed values consist of the joint information of the robotic manipulator, specifically the sine and cosine of the joint position and the joint velocity. By replacing the joint position $q_{i}$ with $\sin(q_{i})$ and $\cos(q_{i})$, the discontinuous position measurements can be expressed through continuous two-dimensional parameterization, while constraining the joint position to [−1,1]. This approach greatly reduces the complexity of the DRL training process and facilitates training convergence. For $S_{2}$, the observed values include the joint position error and the error integral. The error integral reflects the long-term deviation of the system, and its inclusion helps to reduce the steady-state error. For $S_{3}$, the observed values comprise the action at the previous time step and the disturbances observed at each joint. Observing the action helps prevent training from converging to extreme values and enhances the stability of the strategy, whereas observing the disturbances enables rapid assessment of the tracking performance and timely adjustment of the strategy. It should be noted that the observations are normalized to prevent gradient explosion.

#### 3.4.2. Action Space Design

Based on the designed nonlinear disturbance observer-based terminal sliding mode control method, the action space of DRL is defined as the proportional gain, integral gain, and robust gain of NDONFT, that is $a=(K_{p1},K_{i1},K_{r1},K_{p2},K_{i2},K_{r2},K_{p3},K_{i3},K_{r3})$.

**Figure 4:**
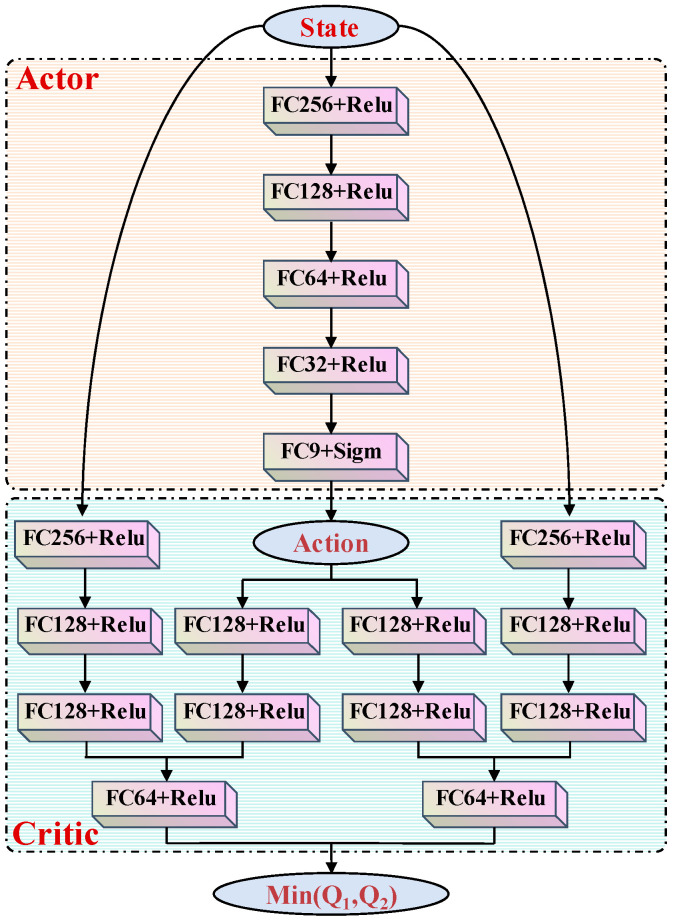
The structure of the actor network and critic network.

The network structure of the critic and actor in TD3NDONFT is shown in Figure 4. The two critic networks share the same structure, with an input layer consisting of the state vector $s_{t}$ and the action vector $a_{t}$. The four intermediate hidden layers are fully connected with 256, 128, 128, and 64 nodes, respectively. The output layer produces a one-dimensional Q-value evaluating the action. The input layer of the actor network is the state vector $s_{t}$. The intermediate hidden layers are fully connected with 256, 128, 64, and 32 nodes, respectively. The output layer is a parameter vector representing the action vector $a_{t}$. According to the action parameter constraints, the output parameters are limited by a sigmoid layer to restrict actions within (0, 1). Additionally, the actions are scaled up by applying gains during simulation.

#### 3.4.3. Reward Function Design

During DRL training, the reward function plays a crucial role in effectively guiding the agent to meet task requirements. To address the problem of sparse rewards, a composite reward function integrating trajectory tracking control is designed, consisting of the following components.

(1) Tracking error: To reduce the tracking error of the robotic manipulator, a distance-based reward is established based on the distance between the current position and the desired position. The tracking error includes position tracking error and velocity tracking error, the reward is expressed as follows:

(46) $$r_{e}=\left\{ \begin{array}{l} c_{1}\times \sum_{i=1}^{n}e^{-c_{e}\times \left|q_{di}(t)-q_{i}(t)\right|}+c_{2}\times \sum_{i=1}^{n}\left|q_{di}(t)-q_{i}(t)\right|\\ c_{3}\times \sum_{i=1}^{n}e^{-c_{e}\times \left|{\dot{q}}_{di}(t)-{\dot{q}}_{i}(t)\right|}+c_{4}\times \sum_{i=1}^{n}\left|{\dot{q}}_{di}(t)-{\dot{q}}_{i}(t)\right| \end{array}\right.$$

where $c_{1}$, $c_{2}$, $c_{3}$ and $c_{4}$ are the weights assigned to the penalty components of position and velocity. As shown in Equation (46), when the position and velocity tracking errors approach zero, a higher reward is returned; when these errors are large, a lower reward is received.

(2) Influence of the previous time step: To reduce the influence of the previous control input on the current step and prevent actions from approaching extreme values that cause the agent to converge to a local optimum, the following equation is incorporated into the reward function:

(47) $$r_{t}=c_{5}\times \sum_{i=1}^{n}a_{i}{\left(t-1\right)}^{2}$$

where $c_{5}$ is the weight assigned to penalizing the action.

(3) Boundary reward: when the joint position exceeds a predefined limit, the following reward function applies:

(48) $$r_{s}=\left\{\begin{array}{ll} 0, & if|q_{i}(t)|<\psi \\ -100, & other \end{array}\right.$$

Through all the above designs, the reward for robotic manipulator trajectory tracking is summarized as $r=r_{e}+r_{t}+r_{s}$.

Based on the above design, a NFTSMC method for robotic manipulators combining NDO and TD3 algorithm is proposed. The NDO estimates and compensates for lumped disturbances online, while the TD3 algorithm achieves parameter adaptation in terminal sliding mode control. Before training, random parameters are first used to initialize the evaluate critic network, evaluate actor network, target critic network, target actor network, and parameters in the experience replay buffer. Then, the robotic manipulator dynamics model is loaded as the environment. To better improve the adaptive performance of the controller, the desired trajectory of the robotic manipulator is randomly initialized at the beginning of each episode. At each time step, the current state $s_{t}$ of the robotic manipulator is input into the target network to obtain the action vector $a_{t}$. The action vector $a_{t}$ is proportionally scaled and input into the sliding mode controller together with the disturbance estimate $\hat{F}$ observed by the NDO. The reward $r_{t}$ at the current time step is calculated based on data including position error and velocity error, and then the next state $s_{t+1}$ of the robotic manipulator is obtained. The tuple data $\left(s_{t},a_{t},r_{t},s_{t+1}\right)$ generated during this process is stored in the experience replay buffer. When the buffer reaches the predefined size, a mini-batch of tuple data is randomly sampled to update the six NNs mentioned above.

## 4. Simulation Studies

The recommended workstation configuration and simulation environment for this study are as follows: operating system, Windows 11; processor, intel(R) Core (TM) i7-10700 CPU @ 2.90 GHz; RAM, 32.0 GB; 64-bit operating system; simulation software, MATLAB, Version: R2024a. We also used the Simulink environment and Simscape toolbox in MATLAB. The development code is in MATLAB language.

**Figure 5:**
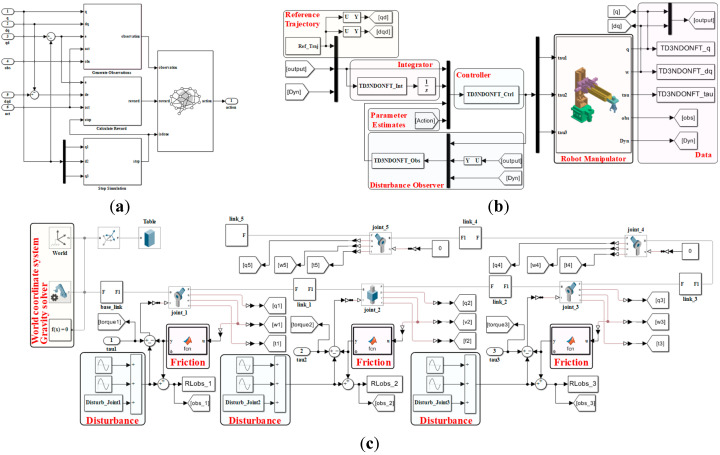
Simulation model of the robotic manipulator. (**a**) DRL framework; (**b**) TD3NDONFT control program construction; (**c**) 5-DOF robotic manipulator constructed with Simscape.

In this section, in order to verify the effectiveness of the proposed algorithm, the two end joints of the designed robotic manipulator are fixed, and simulation is performed using the initial three joints as an example. Simulink and Simscape toolboxes are used to build a simulation model of the controlled robotic manipulator. The simulation results validate the effectiveness of the designed control algorithm. The simulation model of the designed robotic manipulator trajectory tracking control algorithm is shown in Figure 5.

During the training process, the desired trajectory of the robotic manipulator is set as shown in Equation (49). Since the initial position of the prismatic joint is set at the base during dynamic modeling, an initial position offset exists for $q_{2d}$.

(49) $$q_{d}=\left[\begin{array}{c} q_{1d}\\ q_{2d}\\ q_{3d} \end{array}\right]=\left[\begin{array}{c} rand\sin\left(t-\frac{\pi }{8}\right)\\ 0.2+0.06rand\sin\left(t-\frac{\pi }{8}\right)\\ rand\sin\left(t-\frac{\pi }{8}\right) \end{array}\right]$$

The initial joint positions of the three joints for the robotic manipulator are set to $\begin{array}{c} q_{1}(0)=0\mathrm{rad},q_{2}(0)=0.2m, \end{array}q_{3}(0)=0\mathrm{rad}$. The initial desired positions can be calculated using Equation (49). The physical parameters of the designed manipulator are listed in Table 2.

To demonstrate the robustness of the proposed control scheme, modeling errors, friction, and external disturbances are introduced into the robotic manipulator system. In this study, 80% of the dynamic parameters correspond to the nominal model, while 20% represent modeling errors in the system dynamics.

(50) $$\left\{\begin{array}{l} M_{0}(q)=0.8M(q),\Delta M(q)=0.2M(q)\\ C_{0}(q,\dot{q})=0.8C(q,\dot{q}),\Delta C(q,\dot{q})=0.2C(q,\dot{q})\\ G_{0}(q)=0.8G(q),\Delta G(q)=0.2G(q) \end{array}\right.$$

The disturbance $\tau_{d}$ acting on each joint of the robotic manipulator is assumed to be time-varying and consists of three components:

(51) $$\begin{array}{cc} \tau_{d} & =\tau_{d1}+\tau_{d2}+\tau_{d3}\\ & =\left[5\sin(t)+\sin(5t)\right]+\left[0.5\sin(\dot{q})+0.5\dot{q}\right]+\left[10\sin(t-5)\right] \end{array}$$

where $\tau_{d1}$ is a small time-varying disturbance, $\tau_{d2}$ is joint friction, and $\tau_{d3}$ is an unknown large time-varying disturbance introduced at 5s to verify the adaptability and robustness of the proposed control method.

The proposed algorithm is compared with non-singular fast terminal sliding mode control based on disturbance observer (NDONFT), non-singular fast terminal sliding mode control (NFTSM), NDOPID, and PID controllers to verify its effectiveness.

(1) PID: Proportional-integral-derivative controller, the control law is as follows:

(52) $$\tau =k_{p}e+k_{i}\int_{0}^{t}edt+k_{d}\dot{e}$$

For the PID controller, $k_{p},k_{i},k_{d}$ represent the proportional, integral, and derivative coefficients, respectively. The selection of parameters mainly relies on experience and tuning. The selection principle is to minimize oscillations during the initial position tracking phase while ensuring trajectory tracking accuracy.

(2) NDOPID: PID controller incorporating the nonlinear disturbance observer; the control law is as follows:

(53) $$\tau =k_{p}e+k_{i}\int_{0}^{t}edt+k_{d}\dot{e}-\hat{F}$$

For the NDOPID controller, in order to ensure the fairness of the comparison, its basic parameters are selected based on those of the PID controller. The disturbance observer estimates disturbances to compensate the PID control.

(3) NFTSM: Non-singular fast terminal sliding mode control excluding disturbance compensation observed by the NDO and parameter adaptation via the TD3 algorithm. The control law is as follows:

(54) $$\tau =M_{0}(q){\ddot{q}}_{r}+C_{0}(q,\dot{q}){\dot{q}}_{r}+G_{0}(q)+K_{p}r+K_{i}\int_{0}^{t}rdt+K_{r}sat(r)$$

To ensure fairness in the controller comparison, the sliding mode coefficient α,β,p,q is set equal to those in the NDONFT and TD3NDONFT controllers, and the coefficient $K_{p},K_{i},K_{r}$ is set equal to that in the NDONFT controller.

(4) NDONFT: Non-singular fast terminal sliding mode controller based on the NDO, excluding the adaptive strategy of TD3. The control law is as follows:

(55) $$\tau =M_{0}(q){\ddot{q}}_{r}+C_{0}(q,\dot{q}){\dot{q}}_{r}+G_{0}(q)+K_{p}r+K_{i}\int_{0}^{t}rdt+K_{r}sat(r)-\hat{F}$$

For the NDONFT controller, $X^{-1}$ is an invertible matrix used to calculate the observer gain matrix and auxiliary variables. The same value of $X^{-1}$ is used across controllers involving disturbance observers.

(5) TD3NDONFT: Non-singular fast terminal sliding mode control based on TD3 and NDO, with adaptive adjustment of control parameters achieved via the TD3 algorithm. The control law is as follows:

(56) $$\tau =M_{0}(q){\ddot{q}}_{r}+C_{0}(q,\dot{q}){\dot{q}}_{r}+G_{0}(q)+K_{pt}r+K_{it}\int_{0}^{t}rdt+K_{rt}sat(r)-\hat{F}$$

For the TD3NDONFT controller, the adaptive parameters $K_{pt},K_{it},K_{rt}$ are obtained via the TD3 algorithm. Sensitivity analysis of the controller parameters is conducted to set the range of parameter variation during training. The training parameters of the TD3 algorithm are listed in Table 3.

The parameter settings of the five control laws mentioned above are listed in Table 4.

**Figure 6:**
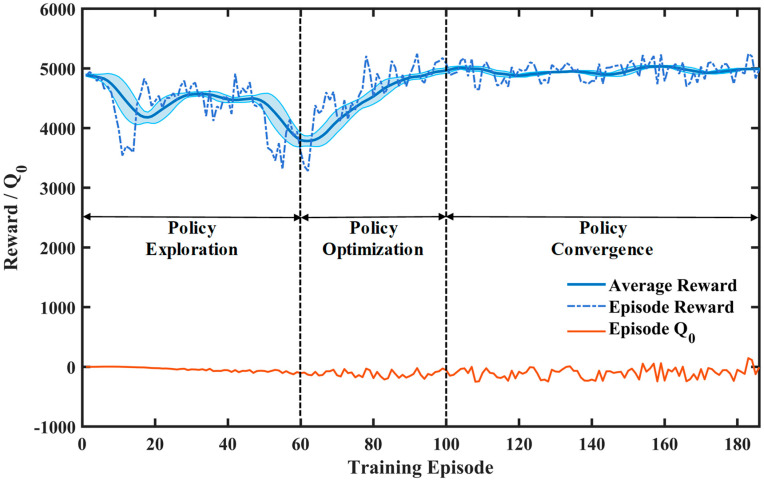
Reward of the Twin Delayed Deterministic Policy Gradient algorithm.

Figure 6 shows the reward curve of the TD3 algorithm presented in this paper, which serves as a key indicator for evaluating the training effectiveness of DRL algorithms. During the policy exploration phase, the curve shows considerable volatility with a low and unstable average reward level, indicating that the strategy of the agent has not yet effectively explored action sequences with higher rewards and remains in an exploratory learning phase. Subsequently, the performance enters an optimization phase, with the average reward showing an overall upward trend and volatility gradually decreasing, indicating the strategy begins to learn more effective action selection mechanisms, leading to significant performance improvement. In the strategy convergence phase, the average reward tends to stabilize and the standard deviation converges to a low level, indicating the strategy has reached a stable convergence state, demonstrating strong robustness and generalization capability.

To further compare the dynamic characteristics of the five controllers, this paper evaluates controller performance using the mean absolute error (MAE) of joint position and joint velocity, the integral absolute error (IAE), and the integral time absolute error (ITAE).

(57) $$\begin{array}{l} MAE=\frac{1}{t_{f}}\int_{0}^{t_{f}}\left|e(t)\right|dt\\ IAE=\int_{0}^{t_{f}}\left|e(t)\right|dt\\ ITAE=\int_{0}^{t_{f}}t\cdot \left|e(t)\right|dt \end{array}$$

where $t_{f}$ represents the total time.

**Figure 7:**
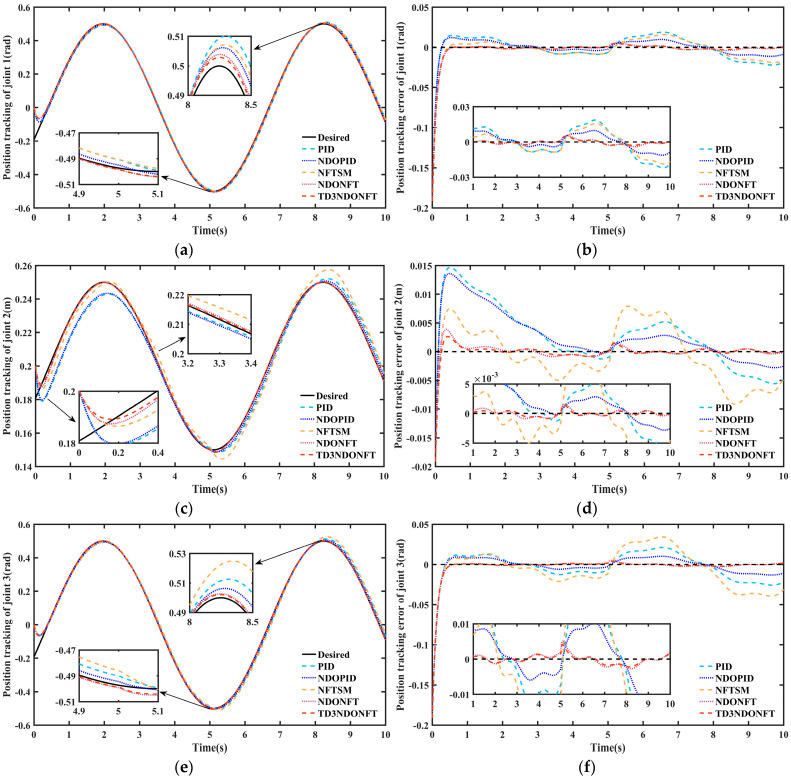
Joint position tracking and position tracking error of robotic manipulator. (**a**) Position tracking comparison of joint 1; (**b**) Position tracking error comparison of joint 1; (**c**) Position tracking comparison of joint 2; (**d**) Position tracking error comparison of joint 2; (**e**) Position tracking comparison of joint 3; (**f**) Position tracking error comparison of joint 3.

After the training is completed, the desired trajectories of joint 1 and joint 3 of the robotic manipulator are set to $q_{1d}=q_{3d}=0.5\sin\left(t-\pi /8\right)$, and the desired trajectory of joint 2 is set to $q_{2d}=0.2+0.05\sin\left(t-\pi /8\right)$ for simulation. Figure 7 shows the simulation results of joint position tracking and tracking errors. All five algorithms ensure trajectory tracking within a certain error range before and after sudden disturbances. Under different control methods, the tracking errors of each joint fall within the ranges of [−0.03, 0.03] rad, [−0.01, 0.015] m, and [−0.04, 0.04] rad, respectively, demonstrating a certain degree of disturbance rejection under sudden disturbances. In the initial stage, the PID and NDOPID control methods exhibit significant overshoot, while the NFTSM, NDONFT, and TD3NDONFT controllers track the desired trajectory at a relatively fast speed. At 5 s, the joints experience a large unknown sudden disturbance. The maximum position tracking errors of the three joints after the disturbance are 0.0048 rad, 0.0014 m, and 0.0041 rad for the proposed TD3NDONFT algorithm; 0.0054 rad, 0.0016 m, and 0.0051 m for the NDONFT method; and 0.0188 rad, 0.0092 m, and 0.0393 rad for the NFTSM method. The results indicate that the proposed control strategy better suppresses external unknown time-varying disturbances.

The MAE indicators for position tracking under different control strategies are presented in Table 5. Both before and after applying sudden disturbances, the proposed control method demonstrates superior tracking performance. During 0–10 s, the MAE of joint positions using the TD3NDONFT algorithm reduce by 7.14%, 19.94%, and 6.14% compared to NDONFT, by 64.58%, 88.06%, and 84.53% compared to NFTSM, by 53.35%, 85.40%, and 63.43% compared to NDOPID, and by 70.60%, 88.48%, and 77.70% compared to PID, respectively. The NDONFT algorithm reduces the MAE by 49.76%, 81.77%, and 61.04% compared to NDOPID.

**Figure 8:**
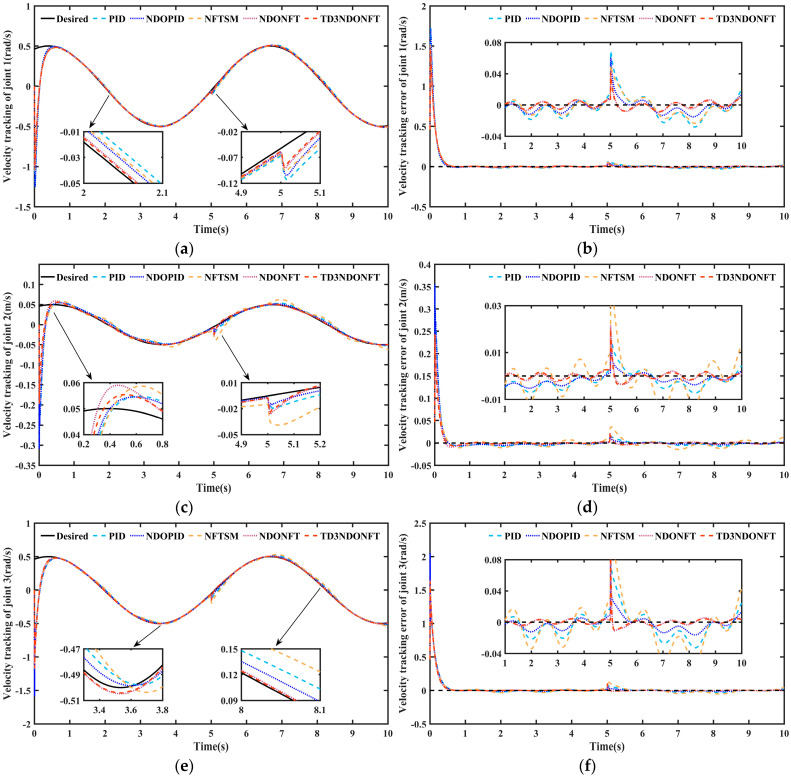
Joint velocity tracking and velocity tracking error of robotic manipulator. (**a**) Velocity tracking comparison of joint 1; (**b**) Velocity tracking error comparison of joint 1; (**c**) Velocity tracking comparison of joint 2; (**d**) Velocity tracking error comparison of joint 2; (**e**) Velocity tracking comparison of joint 3; (**f**) Velocity tracking error comparison of joint 3.

Figure 8 shows the joint velocity tracking of the robotic manipulator and its error. In the initial stage, the velocity tracking exhibits a distinct reverse sudden change. This occurs because the initial state of the robotic manipulator does not match the desired state, which causes the controller to overcompensate at this stage and subsequently enables it to quickly track the desired velocity. Analysis of the velocity tracking error in Figure 8 indicates that although the system velocity experiences significant disturbance after a sudden disturbance, all five control methods converge to the desired velocity. The maximum velocity tracking error of joint 1 after a sudden disturbance using the proposed TD3NDONFT algorithm is 0.0409 rad. 0.0436 rad using the NDONFT method, 0.0506 rad using the NFTSM method, 0.0602 rad using the NDOPID method, and 0.0691 rad using the PID method.

Table 6 shows the MAE performance indicators of velocity tracking under different control methods. Before and after applying sudden disturbances, the algorithm proposed maintains a smaller MAE of velocity after reaching the steady state. Within 0–10 s, the MAE of the velocity of each joint using the TD3NDONFT algorithm reduces by 1.78%, 9.10%, and 2.11% compared to NDONFT, by 19.26%, 58.01%, and 44.64% compared to NFTSM, by 14.18%, 37.37%, and 17.03% compared to NDOPID, and by 27.18%, 48.67%, and 32.45% compared to PID, respectively. This indicates that the proposed algorithm achieves high velocity tracking accuracy and robustness.

**Figure 9:**
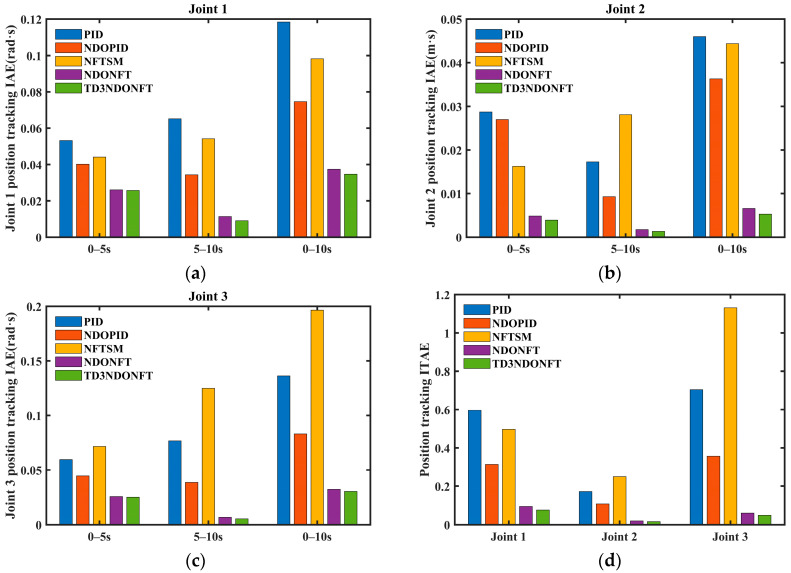
Comparison of IAE and ITAE performance for five position tracking algorithms. (**a**) Joint 1 position tracking IAE; (**b**) Joint 2 position tracking IAE; (**c**) Joint 3 position tracking IAE; (**d**) Position tracking ITAE.

**Figure 10:**
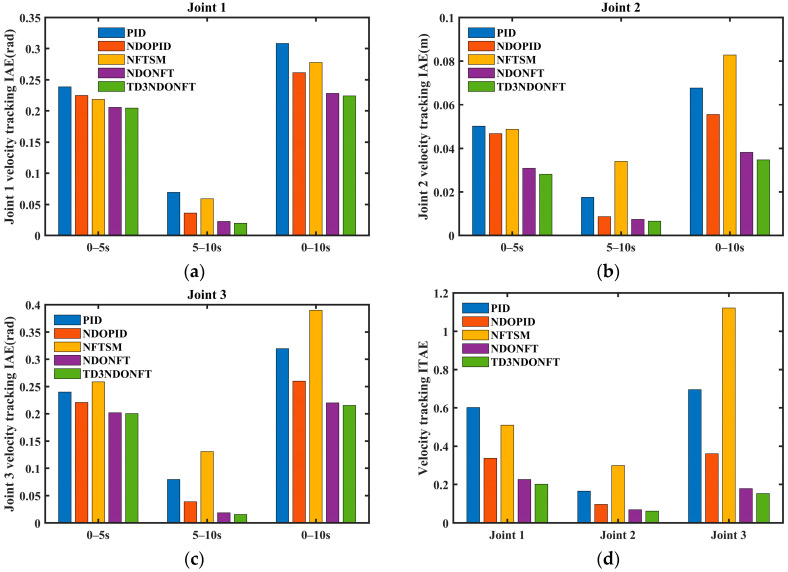
Comparison of IAE and ITAE performance for five velocity tracking algorithms. (**a**) Joint 1 velocity tracking IAE; (**b**) Joint 2 velocity tracking IAE; (**c**) Joint 3 velocity tracking IAE; (**d**) Velocity tracking ITAE.

Figure 9 and Figure 10 show the IAE and ITAE indicators for position and velocity tracking using the five algorithms. The IAE indicators for the three joints of the proposed algorithm are the lowest in the 0–5 s, 5–10 s, and 0–10 s intervals, indicating that the TD3NDONFT algorithm achieves high position and velocity tracking accuracy before and after experiencing unknown time-varying disturbances. Next, the effectiveness of the TD3 adaptive algorithm, nonlinear disturbance observer, and improved non-singular fast terminal sliding mode within the proposed TD3NDONFT algorithm is analyzed.

Within 0–10 s, the TD3NDONFT algorithm reduces the IAE of joint position tracking by 7.16%, 19.97%, and 6.16% compared to NDONFT, and reduces the IAE of velocity tracking by 1.78%, 9.11%, and 2.11%, indicating that introducing the TD3 adaptive algorithm improves the tracking performance of the robotic manipulator. Compared to NFTSM, NDONFT reduces the IAE of position tracking for each joint by 61.91%, 85.11%, and 83.56%, and reduces the IAE of velocity tracking by 17.81%, 53.82%, and 43.47%, respectively. Compared to PID, NDOPID reduces the position tracking IAE by 37.01%, 21.09%, and 39.05%, and the velocity tracking IAE by 15.17%, 18.05%, and 18.60%, indicating the effectiveness of introducing the nonlinear disturbance observer. Compared to NDOPID, NDONFT reduces the position tracking IAE by 49.82%, 81.79%, and 61.11%, and the velocity tracking IAE by 12.63%, 31.11%, and 15.25%, demonstrating the effectiveness of the improved non-singular fast terminal sliding mode. At the same time, the ITAE of position and velocity tracking for the three joints using the TD3NDONFT algorithm is the lowest among the five algorithms.

**Figure 11:**
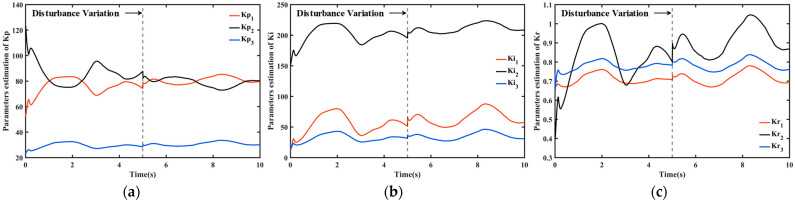
The variation in adaptive parameters. (**a**) Parameter variations of $K_{p}$ at each joint; (**b**) Parameter variations of $K_{i}$ at each joint; (**c**) Parameter variations of $K_{r}$ at each joint.

The adaptive parameters change proposed in this paper are shown in Figure 11. During the variation in adaptive parameters, the proportional gain $K_{p}$ is initially small to generate low joint torque, thereby avoiding excessive initial torque, and then gradually increases to improve joint tracking capability. However, the prismatic joint must overcome its own weight at startup, so a larger initial proportional gain is required. The integral gain $K_{i}$ is larger for the prismatic joint than for the revolute joint to reduce the system steady-state error. Since the nonlinear disturbance observer has estimated most disturbances, only a small robust gain $K_{r}$ is required to compensate the remaining disturbance. At 5 s, when a sudden disturbance occurs, all parameters adjust accordingly to ensure tracking accuracy under unknown time-varying disturbances.

**Figure 12:**
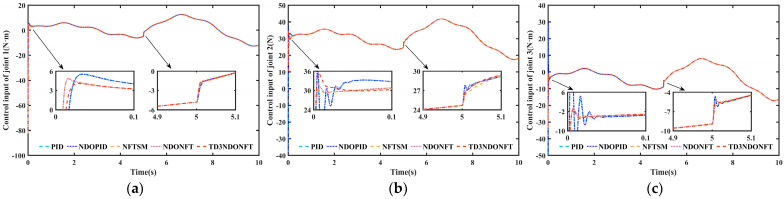
Torque input for robotic manipulator joint control. (**a**) Control input torque of joint 1; (**b**) Control input torque of joint 2; (**c**) Control input torque of joint 3.

Figure 12 shows the control input torque for each joint of the robotic manipulator. In the initial stage, the PID and NDOPID algorithms exhibit large torque oscillations, which may adversely affect the joint actuator and may cause motor driver overload or failure. At 5s, when a sudden disturbance occurs, the torques of all five control methods fluctuate significantly. The torque response under the proposed TD3NDONFT algorithm is smoother, indicating that the method maintains good dynamic performance while ensuring steady-state accuracy.

**Figure 13:**
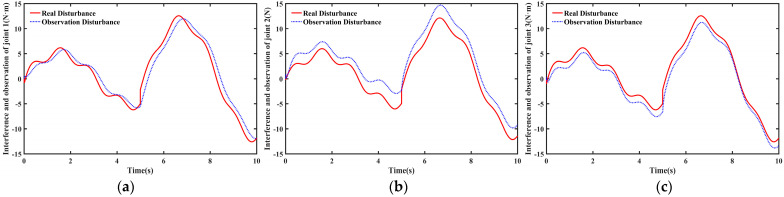
Disturbance observation of each joint for the robotic manipulator. (**a**) Real disturbance and observed disturbance of joint 1; (**b**) Real disturbance and observed disturbance of joint 2; (**c**) Real disturbance and observed disturbance of joint 3.

**Figure 14:**
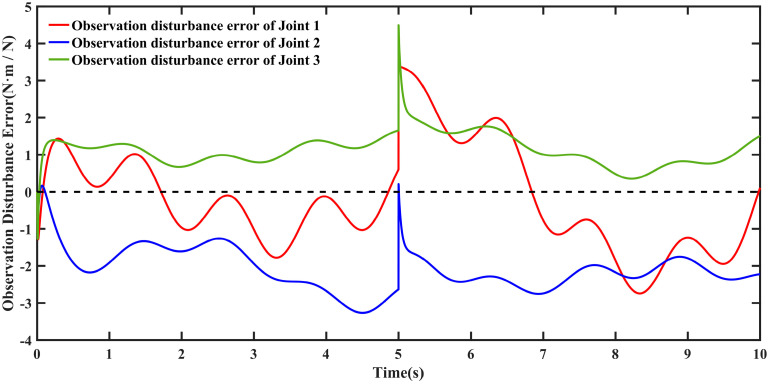
Disturbance observation error of each joint for the robotic manipulator.

Figure 13 shows the actual disturbances during trajectory tracking and the disturbances estimated by the nonlinear disturbance observer. The nonlinear disturbance observer within the proposed TD3NDONFT algorithm effectively estimates disturbances affecting each joint of the robotic manipulator. The joint disturbance estimation errors are shown in Figure 14.

**Figure 15:**
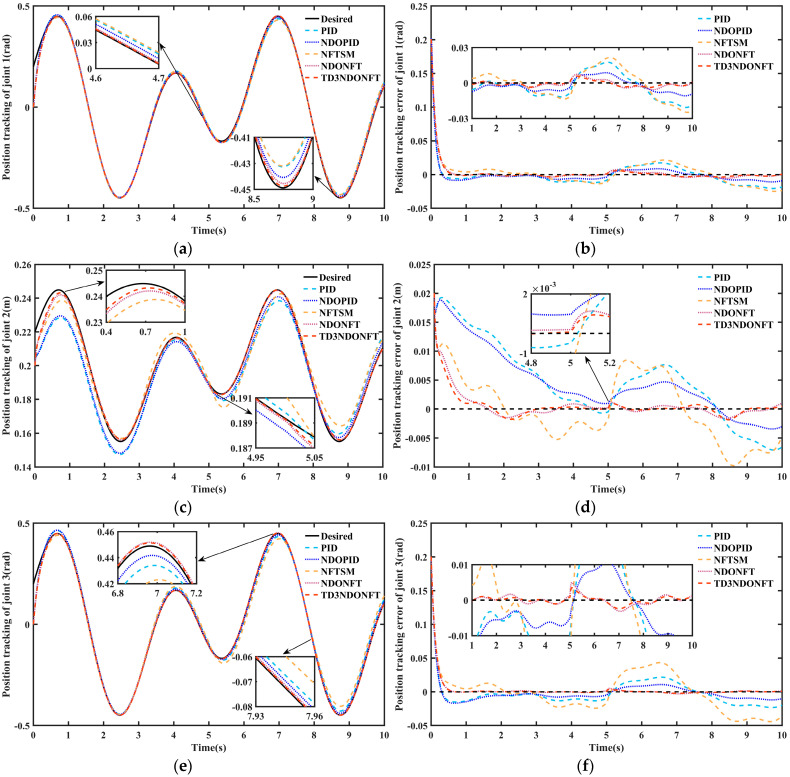
Joint position tracking and position tracking error of robotic manipulator under generalization capability test. (**a**) Position tracking comparison of joint 1; (**b**) Position tracking error comparison of joint 1; (**c**) Position tracking comparison of joint 2; (**d**) Position tracking error comparison of joint 2; (**e**) Position tracking comparison of joint 3; (**f**) Position tracking error comparison of joint 3.

**Figure 16:**
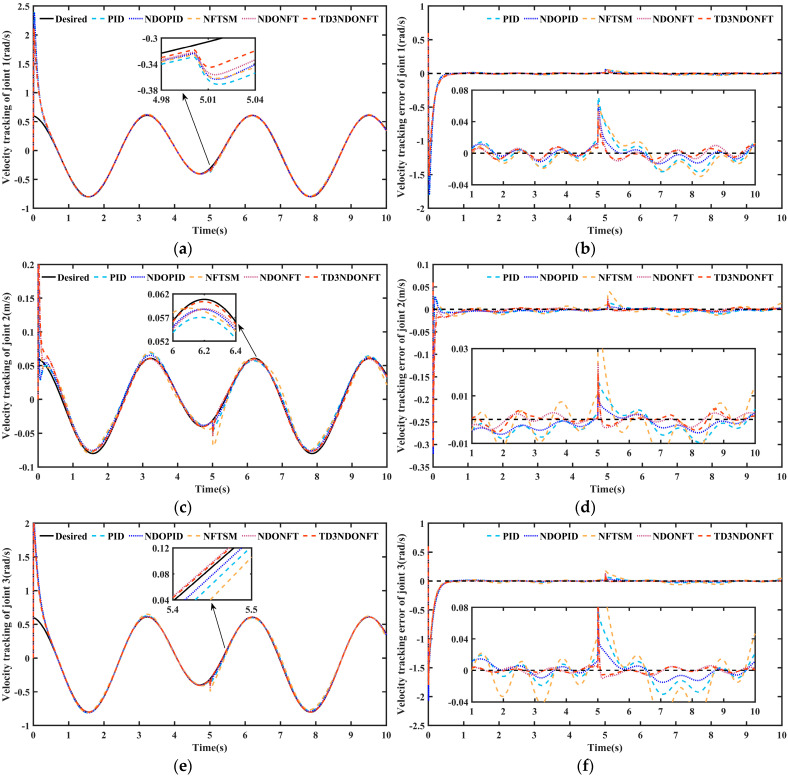
Joint velocity tracking and velocity tracking error of robotic manipulator under generalization capability test. (**a**) Velocity tracking comparison of joint 1; (**b**) Velocity tracking error comparison of joint 1; (**c**) Velocity tracking comparison of joint 2; (**d**) Velocity tracking error comparison of joint 2; (**e**) Velocity tracking comparison of joint 3; (**f**) Velocity tracking error comparison of joint 3.

To further validate the generalization capability of deep reinforcement learning, this paper introduces desired trajectories with more complex dynamic characteristics for testing. The desired trajectories for joint 1 and joint 3 of the robotic arm are set as $q_{1d}=q_{3d}=0.3\sin\left(2t\right)+0.2\cos(t)$, while the desired trajectory for joint 2 is set as $q_{2d}=0.2+0.03\sin\left(2t\right)+0.02\cos(t)$. Figure 15 presents the position tracking curves and corresponding tracking errors of each joint under different control strategies, while Figure 16 illustrates the velocity tracking performance and velocity tracking errors of the joints. Experimental results demonstrate that the proposed method maintains excellent accuracy and robustness even in complex trajectory tracking tasks, fully reflecting its strong generalization performance.

## 5. Conclusions

This paper studies the trajectory tracking control problem of a novel robotic manipulator configuration. The main contributions are as follows:

1. For robotic manipulator system with modeling uncertainties, friction, and unknown external time-varying disturbances, an adaptive non-singular fast terminal sliding mode control strategy based on the Twin Delayed Deep Deterministic policy gradient algorithm and a nonlinear disturbance observer is proposed. Stability and finite-time convergence of the closed-loop system are established via Lyapunov analysis.
2. A nonlinear disturbance observer estimates the lumped uncertainty of the robot manipulator and provides feedforward compensation. Based on boundary layer techniques, the non-singular fast terminal sliding mode is modified to reduce chattering in sliding mode control. Adaptive control of the desired trajectory is achieved using the Twin Delayed Deep Deterministic policy gradient algorithm.
3. Training simulations are conducted using the designed 5-DOF robotic manipulator as an example. Convergence of training is ensured through the design of the observation space and reward function for the Twin Delayed Deep Deterministic policy gradient algorithm. The training process considers trajectory tracking accuracy under sudden disturbances to ensure that the robotic manipulator can handle emergency situations.
4. Using the trained agent, different control strategies are compared. Compared with PID, NDOPID, NFTSM, and NDONFT controllers, TD3NDONFT achieves higher trajectory tracking accuracy, lower MAE, IAE, and ITAE across time intervals, and stronger robustness against sudden disturbances. By randomizing the desired trajectory, the proposed algorithm exhibits improved generalization and attains higher tracking accuracy across different trajectory configurations.

This study provides a new approach for the development of robotic manipulators with novel configurations and the realization of high-precision trajectory tracking control. Although the simulation results are promising, a systematic comparison with other mainstream deep reinforcement learning algorithms is still lacking, and exploring a broader hyperparameter space to obtain more generalizable conclusions also represents a valuable direction for future research. In addition, the performance of robotic manipulators is affected by various factors such as sensor noise and communication delays in practical applications. Future work will focus on establishing an experimental platform to validate the effectiveness of the proposed algorithm and exploring its application in trajectory tracking tasks for other nonlinear systems.

## Figures and Tables

**Table 1:** MDH Parameters of the Robotic Manipulator.

Joints	Type	$d_{i}/\mathrm{mm}$	$q_{i}/\mathrm{rad}$	$a_{i-1}/\mathrm{mm}$	$\alpha_{i-1}/\mathrm{rad}$
Joint 1	revolute	154.55	$q_{1}$	0	0
Joint 2	prismatic	$d_{2}$	0	12.00	0
Joint 3	revolute	0	$q_{3}$	174.46	−π/2
Joint 4	revolute	0	$q_{4}$	389.83	0
Joint 5	revolute	0	$q_{5}$	70.00	π/2

**Table 2:** Physical parameters of the robotic manipulator.

Symbol	Definition	Value
M_1_	Mass of Link 1	2.98 kg
M_2_	Mass of Link 2	1.47 kg
M_3_	Mass of Link 3	1.05 kg
M_4_	Mass of Link 4	0.32 kg
M_5_	Mass of Link 5	0.18 kg
g	Gravity acceleration	9.806 m/s^2^

**Table 3:** Training parameters of the TD3 algorithm.

Parameter Symbol	Value
Actor network learning rate	0.0001
Critic network learning rate	0.0001
Sampling step size	0.01
Discount factor	0.995
Experience replay size	1,000,000
Minibatch size	128
Noise variance	0.1
Threshold of gradient	1
Max Step	1000

**Table 4:** Parameter settings in the control law.

Control Law	Parameter Settings
PID	$k_{p}=diag(500,2000,400),k_{i}=diag(300,1000,300),k_{d}=diag(50,200,50)$
NDOPID	$\begin{array}{l} k_{p}=diag(500,2000,400),k_{i}=diag(300,1000,300),k_{d}=diag(50,200,50)\\ X^{-1}=diag(0.5251,0.0927,0.6277) \end{array}$
NFTSM	$\begin{array}{l} K_{p}=diag(70,75,25),K_{i}=diag(40,150,25),K_{r}=diag(0.7,0.7,0.7)\\ \alpha =8,\beta =2,p=5,q=3 \end{array}$
NDONFT	$\begin{array}{l} K_{p}=diag(70,75,25),K_{i}=diag(40,150,25),K_{r}=diag(0.7,0.7,0.7)\\ \alpha =8,\beta =2,p=5,q=3,X^{-1}=diag(0.5251,0.0927,0.6277) \end{array}$
TD3NDONFT	$\begin{array}{l} \alpha =8,\beta =2,p=5,q=3,X^{-1}=diag(0.5251,0.0927,0.6277)\\ c_{1}=c_{3}=1,c_{2}=c_{4}=-5,c_{5}=-0.3,c_{e}=-10 \end{array}$

**Table 5:** Position tracking mean absolute error evaluation indexes.

Controllers	Joints	Position Tracking Mean Absolute Error		
		0–5 (s)	5–10 (s)	0–10 (s)
PID	Joint 1	1.064 × 10^−2^	1.305 × 10^−2^	1.185 × 10^−2^
	Joint 2	5.745 × 10^−3^	3.457 × 10^−3^	4.601 × 10^−3^
	Joint 3	1.192 × 10^−2^	1.535 × 10^−2^	1.364 × 10^−2^
NDOPID	Joint 1	8.054 × 10^−3^	6.879 × 10^−3^	7.467 × 10^−3^
	Joint 2	5.403 × 10^−3^	1.858 × 10^−3^	3.631 × 10^−3^
	Joint 3	8.919 × 10^−3^	7.714 × 10^−3^	8.317 × 10^−3^
NFTSM	Joint 1	8.845 × 10^−3^	1.082 × 10^−2^	9.834 × 10^−3^
	Joint 2	3.256 × 10^−3^	5.624 × 10^−3^	4.440 × 10^−3^
	Joint 3	1.434 × 10^−2^	2.499 × 10^−2^	1.966 × 10^−2^
NDONFT	Joint 1	5.219 × 10^−3^	2.283 × 10^−3^	3.751 × 10^−3^
	Joint 2	9.744 × 10^−4^	3.497 × 10^−4^	6.621 × 10^−4^
	Joint 3	5.147 × 10^−3^	1.333 × 10^−3^	3.240 × 10^−3^
TD3NDONFT	Joint 1	5.152 × 10^−3^	1.814 × 10^−3^	3.483 × 10^−3^
	Joint 2	7.866 × 10^−4^	2.734 × 10^−4^	5.301 × 10^−4^
	Joint 3	5.029 × 10^−3^	1.053 × 10^−3^	3.041 × 10^−3^

**Table 6:** Velocity tracking Mean absolute error evaluation indexes.

Controllers	Joints	Velocity Tracking Mean Absolute Error		
		0–5 (s)	5–10 (s)	0–10 (s)
PID	Joint 1	4.781 × 10^−2^	1.385 × 10^−2^	3.083 × 10^−2^
	Joint 2	1.004 × 10^−2^	3.508 × 10^−3^	6.774 × 10^−3^
	Joint 3	4.803 × 10^−2^	1.589 × 10^−2^	3.196 × 10^−2^
NDOPID	Joint 1	4.506 × 10^−2^	7.262 × 10^−3^	2.616 × 10^−2^
	Joint 2	9.352 × 10^−3^	1.750 × 10^−3^	5.551 × 10^−3^
	Joint 3	4.428 × 10^−2^	7.757 × 10^−3^	2.602 × 10^−2^
NFTSM	Joint 1	4.380 × 10^−2^	1.182 × 10^−2^	2.781 × 10^−2^
	Joint 2	9.749 × 10^−3^	6.813 × 10^−3^	8.281 × 10^−3^
	Joint 3	5.179 × 10^−2^	2.620 × 10^−2^	3.900 × 10^−2^
NDONFT	Joint 1	4.116 × 10^−2^	4.554 × 10^−3^	2.286 × 10^−2^
	Joint 2	6.158 × 10^−3^	1.491 × 10^−3^	3.825 × 10^−3^
	Joint 3	4.040 × 10^−2^	3.702 × 10^−3^	2.205 × 10^−2^
TD3NDONFT	Joint 1	4.091 × 10^−2^	3.986 × 10^−3^	2.245 × 10^−2^
	Joint 2	5.619 × 10^−3^	1.334 × 10^−3^	3.477 × 10^−3^
	Joint 3	4.009 × 10^−2^	3.083 × 10^−3^	2.159 × 10^−2^

## Data Availability

The original contributions presented in this study are included in the article. Further inquiries can be directed to the corresponding author.
